# Translation and comprehensive validation of the Neurological Outcome Scale for Traumatic Brain Injury (NOS-TBI) into Brazilian Portuguese

**DOI:** 10.1055/s-0046-1829021

**Published:** 2026-10-06

**Authors:** Karina T. Weber, Luciana B. M. Alves, Monica C. Braga, Suleimy C. Mazin, Brunna R. Pileggi, Millene R. Camilo, Viviane A. Guimaraes, Clara M. A. Barreira, Frederico Alessio-Alves, Aline S. Carvalho, Aline C. Pacheco-Castilho, João P. Leite, Dylan J. Edwards, Octavio M. Pontes-Neto, Taiza G. S. Edwards

**Affiliations:** 1 Universidade de São Paulo, Faculdade de Medicina de Ribeirão Preto, Departamento de Neurociências e Ciências do Comportamento, Ribeirão Preto SP, Brazil.; 2 Thomas Jefferson University, Jefferson Moss Rehabilitation Research Institute, Elkins Park PA, United States.; 3 Edith Cowan University, School of Biomedical and Health Sciences, Exercise Medicine Research Institute, Perth WA, Australia.; 4 University of Western Australia, School of Health and Clinical Sciences, Perth WA, Australia.

**Keywords:** Brain Injuries, Traumatic, Validation Study, Outcome Assessment, Functional Outcome

## Abstract

**Background:**

Traumatic brain injury (TBI) is a leading cause of disability in Brazil and worldwide. National and multinational clinical trials in TBI require global standardization of outcome measures. The original English version of the Neurological Outcome Scale for TBI (NOS-TBI) is a valid and valuable neurological assessment tool. However, NOS-TBI has not yet been validated in Brazilian Portuguese.

**Objective:**

To perform the translation, cultural adaptation, and validation of the NOS-TBI in a Brazilian cohort of individuals with TBI.

**Methods:**

We conducted the translation and cultural adaptation of the NOS-TBI scale to Brazilian Portuguese, followed by an analysis of psychometric properties: internal consistency; interrater reliability; concurrent validity in relation to Revised Trauma Score (RTS) and Glasgow Coma Scale (GCS); and convergent validity in relation to Functional Independence Measure (FIM), and Extended Glasgow Outcome Scale (GOS-E). We assessed 255 subjects with TBI, age 42 ± 17.9 years, male-to-female ratio of 4.2:1. The interrater reliability analysis was conducted in a subcohort of 136 subjects, 85.3% male, aged 40.3 ± 17.5 years.

**Results:**

The Brazilian Portuguese NOS-TBI showed excellent consistency and interrater reliability. The intraclass correlation coefficient (ICC) revealed excellent interrater agreement. The concurrent validity showed strong correlations of the NOS-TBI with both GCS and RTS at admission. For convergent validity, moderate correlations were observed for both GOS-E and FIM.

**Conclusion:**

The Brazilian Portuguese NOS-TBI is valid, reliable, and can be used in clinical practice and scientific studies. Globally, developing relevant and reliable clinical scales in different languages, such as the NOS-TBI, can facilitate multinational studies in the field.

## INTRODUCTION

Traumatic brain injury (TBI) is a major public health challenge, imposing substantial healthcare and societal costs that require coordinated efforts on a global basis. 1 The urgent need for more effective treatments also highlights the necessity for a standardized framework for studying the effects of novel therapies, 1 including outcome measures with cross-cultural validity. Consequently, selecting appropriate scales to stratify patients and identify meaningful clinical changes is a key strategy for improving clinical trials and observational studies. However, despite Brazil's high TBI prevalence and significant contributions to the scientific literature, there is a lack of validated clinical and functional assessment tools for Brazilian Portuguese.

While the Glasgow Coma Scale (GCS) is globally accepted for assessing TBI severity and acute neurological deterioration, it fails to capture several specific deficits common after TBI, such as motor weakness, somatosensory loss, language dysfunction, and motor incoordination. Therefore, comprehensive instruments are needed to better assess the multifactorial neurological consequences of TBI. 2 The Neurological Outcome Scale for TBI (NOS-TBI) was developed to address this need. Based on the National Institutes of Health Stroke Scale (NIHSS), an essential scale in stroke care, the NOS-TBI assesses the full spectrum of neurological deficits following brain injury. Developed by Wilde et al. 2 5 , the English version has demonstrated excellent psychometric properties. 2 3 4 5

The NOS-TBI is recommended by the US National Institute of Neurological Disorders and Stroke (NINDS) as one of the common data elements for TBI investigation 6 and is valuable for lesion severity stratification and clinical data quantification. 2 7 8 However, to date, the NOS-TBI has not been validated in Brazilian Portuguese. The absence of a culturally adapted and psychometrically robust version impedes the standardization of clinical assessments, compromises data comparability across services, and restricts the generation of high-quality national evidence, representing a significant gap for both local practice and research.

Therefore, this study aimed to translate, cross-culturally adapt, and psychometrically validate the NOS-TBI for Brazilian Portuguese, including assessments of internal consistency, interrater reliability, and evidence of concurrent and convergent validity. 9

## METHODS

### Study outline

This comprehensive validation study was conducted as part of a large systematic cohort that prospectively enrolled consecutive patients diagnosed with TBI between January 7, 2014, and May 7, 2015. The study was conducted following the Helsinki Declaration and approved by our institution's Ethics and Research Committee (IRB number 15772/2011). This study followed the Consensus-based Standards for the Selection of Health Measurement Instruments (COSMIN) guidelines.

### Patients and setting

Inclusion criteria for the present analyses were age greater than or equal to 18 years, a confirmed diagnosis of TBI based on clinical and/or neuroimaging examinations, onset of symptoms within 24 hours of admission, participation in the 6-month follow-up, and informed consent. The exclusion criteria were a life expectancy of less than 1 year due to other clinical conditions or a previous neurological disease with a significant functional impairment that would impact prognosis (modified Rankin Scale [mRS] > 2).

### Translation and cross-cultural adaptation of the NOS-TBI into Brazilian Portuguese

The NOS-TBI measure was based on the NIHSS as a guide adjusted for the TBI setting and suitable for both clinical and research purposes. 10 As a result, there are similarities with the NIHSS and additional specific and relevant items for TBI, such as hearing and smell capabilities, pupillary response, lateralization of the deficit (right or left side), and the supplementary items gait, ataxia, and limb ataxia. 11 Thus, the scale comprises 15 neurological items (2 being considered supplementary and 8 having subitems), allowing the evaluation of essential elements during a neurological evaluation. Nontestable (“NT”) is an acceptable answer for some items, as it is not computed in the total score. 2

After the consent for the use of NOS-TBI from the developers, the following steps were taken:

#### *Step I: translation and cross-cultural adaptation to Portuguese*

Two independent Brazilian English teachers who were aware of the objective of this research translated the English version of the NOS-TBI into Brazilian Portuguese. The translators engaged in conceptual emphasis rather than a literal translation. The two versions were then compared and combined by the translators and coordinators of the study. In case of disparities, modifications were made until a consensus was reached regarding the initial versions, which was termed the 'first Brazilian Portuguese version'.

The supplementary material used for aphasia evaluation (figures and phrases) was obtained from the validated NHISS Brazilian Portuguese adapted version. 12 The olfactory function was tested using a standardized Sniffin Sticks kit of olfactory pens (Burghardt Messtechnik GmbH), which allowed the patient to identify four different odors. 11 The odors from the original scale were orange, licorice, vanilla, and cinnamon. However, they were culturally adapted to a Brazilian context, into orange, cinnamon, coffee, and banana, after sample testing in 50 healthy individuals.

#### *Step II: back-translation*

Two native English-speaking translators who had not participated in the previous step performed the back-translation from Brazilian Portuguese to English. The translations were then compared with the original instrument. All discrepancies were documented and analyzed by a committee until a conceptual equivalence with the original version, which was considered the 'second Brazilian Portuguese version'.

#### *Step III: instrument pretest – evaluation of cultural equivalence*

The instrument was applied to 10 patients with TBI admitted to the Emergency Unit by a physiotherapist and a physician. At the end of each test, assessors described the difficulties in using the scale. After analyzing each NOS-TBI item, the necessary adaptations were made to the Brazilian context, aiming not to significantly alter the structure or the evaluation properties of the questions and to maintain the instrument's original capabilities. Thus, the final Brazilian Portuguese version was attained.

### Assessment of the Brazilian Portuguese NOS-TBI's performance

All investigators were previously trained on the English NOS-TBI version and certified on the original NIHSS and for all other measurement tools used in this study, including mRS, Revised Trauma Score (RTS), Extended Glasgow Outcome Scale (GOS-E), GCS, and the Functional Independence Measurement (FIM).

The following psychometric properties of the NOS-TBI (Brazilian Portuguese) were investigated:

- Internal consistency is a reliability measurement that addresses how a test item reliably measures the same concept or portion and was analyzed using Cronbach's α.
- Criterion validity evaluates the specific outcome degree of NOS-TBI measurements for each patient. It encompassed the concurrent validity tested by the correlation analysis of the translated scale with GCS and RTS, assessed during the first 24 hours after trauma. The GCS and RTS examiner was blinded to the NOS-TBI score.
- Convergent validity was used to assess whether NOS-TBI can measure the same constructs as FIM and GOS-E. The scales were evaluated 6 months after trauma. The FIM and GOS-E examiner was blinded to the NOS-TBI score.
- Interrater reliability of the translated scale was tested in an inpatient care setting, at the same time, by two independent examiners. The NOS-TBI items were tested consistently in the same order and way by each examiner. While one examiner performed the test, the other observed it. Examiners were not permitted to communicate with each other during the evaluation. Each examiner scored the NOS-TBI independently.

### Validation toolkit

The GCS evaluates the level of consciousness and TBI severity, ranging from 3 (total unawareness and motor inactivity) to 15 points (full awareness and motor activity); scoring ≤ 8 is considered a severe TBI, or coma. 12

The RTS is a prehospital scoring system that measures systemic trauma reaction by evaluating the GCS, systolic blood pressure, and respiratory rate. Each item is scored from 0 to 4, with a maximum final score of 8. Higher scores represent better prognosis and better survival rate. 13

The FIM is an instrument that evaluates one's functional impairment of diverse etiologies. It quantifies the load of help someone requires to perform daily cognitive and motor activities, such as self-care, transfer and moving, bowel and urinary continence, memory, communication, social skills, and problem-solving. Each item may be scored from 1 (“total assistance with helper”) to 7 (“complete independence with no helper”), with a final score ranging from 18 to 126, and higher scores translating into higher independence. 14 15

Finally, the GOS-E is used to evaluate global functional capability after TBI, grading patients with 8 different degrees of functionality, ranging from total recovery (8) to death (1). 8

### Statistical analyses

Epidemiological and clinical data (descriptive analysis) were applied as appropriate. Internal consistency was calculated using Cronbach's Alpha (excellent: α ≥ 0.9; good: 0.9 > α ≥ 0.8; acceptable: 0.8 > α ≥ 0.7; questionable: 0.7 > α ≥ 0.6; poor: 0.6 > α ≥ 0.5; unacceptable: < 0.5). Concurrent validity of NOS-TBI with GCS and RTS was calculated using Spearman's rank-order correlation (r), being considered weak from 0.2 to 0.29; moderate from 0.30 to 0.59; strong from 0.6 to 0.89; and very strong: 0.90 to 1.0. 9 The intraclass correlation coefficient (ICC) may range from 0 to 1 (excellent: ICC ≥ 0.75; good: 0.75 > ICC ≥ 0.6; fair: 0.6 > ICC ≥ 0.4; and poor: ICC < 0.4).

Kappa's coefficient assessed the agreement between each item within the scale, and the expected findings can be rated like ICC. Statistical analyses were performed using SAS for Windows (SAS Institute Inc.), version 9.3. All tests were two-tailed, with significance for all analyses set at *p* < 0.05.

## RESULTS

The study population consisted of a sample of consecutive eligible patients recruited over a 16-month period. During this time, 255 patients with TBI who met the preestablished inclusion criteria were sequentially enrolled. The study population's mean age was 42 ± 17.9 years, and 206 (80.8%) were male, with a male-to-female ratio of 4.2:1. Figure 1 Regarding injury severity, 203 patients (80.4%) had mild, 12 (4.7%) had moderate, and 38 (14.9%) had severe TBI. The mean RTS score was 7.49 ± 1.12, with the worst score being for transport accident and the best for falls. The mean FIM at 6 months after the trauma was 117.21 ± 16.28. The demographic and clinical characteristics are summarized in Tables 1 2 respectively.

**Table 1:** Demographic characteristics of the participants with traumatic brain injury

Variable		Participants (n = 255)
** Mean age (years) ^a^**		42.01 ± 17.86
**Gender: n (%)**	Male	206 (80.8)
	Female	49 (19.2)
**Trauma mechanism: n (%)**	Motor vehicle passenger	121 (47.4)
	Falls	73 (28.6)
	Aggression	30 (11.8)
	Bicycle accidents	15 (5.9)
	Running over	10 (3.9)
	Other	6 (2.3)
**Occupation: n (%)**	Constructor	49 (19.2)
	Handyman	31 (12.2)
	Retired	28 (11.0)
	Driver	24 (9.4)
	Salesperson	19 (7.5)
	Unemployed	15 (5.9)
	Housewife	12 (4.7)
	Car repairer	11(4.3)
	Healthcare employee	8 (3.1)
	Self-employed	8 (3.1)
	Farmer	6 (2.4)
	Student	4 (1.6)
	Other	40 (15.7)
**Years of schooling: n (%)**	Illiterate	7 (2.7)
	1–4	101 (39.6)
	5–8	35 (13.7)
	9–12	91 (35.7)
	>12	21 (8.2)
**Loss of consciousness: n (%)**	Yes	156 (61.9)
	No	99 (38.8)

**Table 2:** Clinical characteristics of the participants with TBI

Variable		Participants (n = 255)
**Clinical status at hospital admission**		
NOS-TBI ^a,b^		13.82 ± 20.09
		4 (1; 15.5)
GCS ^b^	13–15	205 (80.4)
	9–12	12 (4.7)
	8–3	38 (14.9)
**Clinical status 6 months post-TBI**		
NOS-TBI ^a,b^		2.47 ± 3.74
		1 (0; 3)
GCS ^b^	13–15	252 (98.8)
	9–12	3 (1.2)
	8–3	0 (0)
FIM ^a^		117.21 ± 16.28
GOS-E ^b^	Full recovery	60 (23.5)
	Minor physical or mental defect	61 (23.9)
	Some disability*	78 (30.6)
	Lower moderate disability	21 (8.2)
	Upper severe disability	21 (8.2)
	Lower severe disability	14 (5.5)
	Vegetative state	0 (0)

Abbreviations: FIM, Functional Independence Measure; GCS, Glasgow Coma Scale; GOS-E, Extended Glasgow Outcome Scale; NOS-TBI, Neurological Outcome Scale for TBI; RTS, revised trauma score; TBI, traumatic brain injury.

**Notes:**^a^ Mean ± standard deviation; ^b^ absolute number (%); ^c^ median (interquartile range); *can return to some form of employment.

The Brazilian Portuguese version of the NOS-TBI is available in the **Supplementary Material S1** (available at https://www.arquivosdeneuropsiquiatria.org/wp-content/uploads/2026/05/ANP-2026.0164-Supplementary-Material.pdf ). The NOS-TBI internal consistency was α = 0.96 for each intrascale item. The exclusion of some items from the analysis did not substantially affect Cronbach's α. Many items strongly correlated with the total score, although a few items showed poor correlation, suggesting they may contribute to variance and were not redundant with other items. The correlation between items ranged from -0.026 to 0.83, as shown in Table 3 .

**Table 3:** Internal consistency: Cronbach's α coefficient

Deleted variable	Cronbach coefficient α with deleted variable				
	Raw variables		Standardized variables	
	Correlation	α	Correlation	α
**1a**	0.950186	0.968833	0.935505	0.960135
**1b**	0.834813	0.969918	0.831720	0.961030
**1c**	0.941535	0.969397	0.926851	0.960210
**2**	0.149430	0.973080	0.170818	0.966507
**3a**	0.892599	0.969684	0.884071	0.960580
**3b**	0.914221	0.969567	0.903152	0.960415
**4**	0.731350	0.970657	0.715323	0.962022
**5a**	0.932858	0.969442	0.921382	0.960257
**5b**	0.927416	0.969458	0.912974	0.960330
**6a**	-0.007196	0.973620	0.008885	0.967792
**6b**	-0.048724	0.973867	-0.021123	0.968027
**7a**	0.956712	0.969397	0.918365	0.960283
**7b**	0.915392	0.970005	0.874363	0.960663
**8a**	0.938885	0.969790	0.885458	0.960568
**8b**	0.926939	0.969901	0.878526	0.960627
**9a**	0.936890	0.969507	0.914416	0.960317
**9b**	0.930110	0.969545	0.911877	0.960339
**9c**	0.913851	0.969584	0.889665	0.960531
**9d**	0.922812	0.969533	0.903081	0.960415
**10**	0.940349	0.968914	0.941510	0.960083
**11**	0.277253	0.972820	0.487646	0.963929
**12**	0.923338	0.969469	0.917186	0.960293
**13**	0.765206	0.970402	0.765691	0.961594
**14**	1.016786	0.968485	0.843351	0.960930
**15a**	-0.043757	0.973372	-0.012134	0.967957
**15b**	-0.055605	0.973379	-0.026203	0.968067

The concurrent validity of the NOS-TBI total score showed a high degree of agreement with both GCS (r = -0.69, *p* < 0.001) and RTS (r = -0.63, *p* < 0.001) assessed 24 hours after admission in 255 patients. Convergent validity analyses showed a moderate correlation of NOS-TBI with both GOS-E (r = 0.424, *p* < 0.001) and FIM (r = -0.553, *p* < 0.001) assessed at 6 months after the trauma in 255 patients.

Interrater reliability analyses included a convenience sample of 136 TBI patients, 85.3% male, and the mean age was 40.3 ± 17.5 (median: 35, 18–94) years. As for TBI severity, 52 patients (38.2%) were considered severe, 13 (9.6%) were moderate, and 71 (52.2%) were mild. All NOS-TBI items showed weighed Kappa > 0.73 (0.73–1; Table 4 ). The ICC results revealed excellent interrater agreement (0.97, 0.96–0.98). The simple Kappa coefficient was excellent for all items, except for one item (Limb ataxia left) that was considered satisfactory (k = 0.66).

**Table 4:** Interrater reliability for NOS-TBI items

NOS-TBI items		Simple Kappa			Weighted Kappa		
		κ	ASE	95%CI	κ	ASE	95%CI
1b	LOC questions	0.96	0.02	0.92–1.0	0.96	0.02	0.91–1.0
1c	LOC commands	0.92	0.05	0.83–1.0	0.94	0.04	0.85–1.0
2	Gaze	0.78	0.11	0.55–1.0	0.73	0.14	0.45–1.0
3a	Visual field right	0.96	0.03	0.88–1.0	0.97	0.02	0.92–1.0
3b	Visual field left	0.93	0.04	0.84–1.0	0.95	0.02	0.89–1.0
4	Pupillary response	1.0	0.0	1.0–1.0	1.0	0.0	1.0–1.0
5a	Hearing right	0.92	0.05	0.81–1.0	0.94	0.03	0.87–1.0
5b	Hearing left	0.87	0.06	0.74–1.0	0.92	0.04	0.83–1.0
6a	Facial paresis right	0.88	0.06	0.75–1.0	0.91	0.05	0.81–1.0
6b	Facial paresis left	0.83	0.006	0.69–0.96	0.88	00.4	0.76–0.97
7a	Motor RUE	0.92	0.03	0.84–0.99	0.95	0.02	0.89–1.0
7b	Motor LUE	0.89	0.03	0.81–0.97	0.94	0.02	0.89–0.99
8a	Motor RLE	0.94	0.02	0.89–0.97	0.95	0.02	0.90–1.0
8b	Motor LLE	0.90	0.03	0.84–0.97	0.91	0.03	0.85–0.98
9a	Sensory RUE	0.90	0.04	0.80—0.99	0.88	0.06	0.76–1.0
9b	Sensory LUE	0.90	0.05	0.78–0.99	0.91	0.04	0.83–0.99
9c	Sensory RLE	0.88	0.05	0.78–0.99	0.91	0.04	0.83–0.99
9d	Sensory LLE	0.88	0.05	0.78–0.99	0.91	0.04	0.83–0.99
10	Language	0.97	0.02	0.93–1.0	0.96	0.03	0.89–1.0
11	Dysarthria	0.93	0.04	0.85–1.0	0.90	0.06	0.77–1.0
12	Neglect/extinction	0.93	0.04	0.85–1.0	0.95	0.03	0.88–1.0
13	Olfaction	0.91	0.03	0.85–0.98	0.92	0.02	0.87–0.98
14	Gait ataxia	0.97	0.02	0.94–1.0	0.98	0.01	0.96–1.0
15a	Limb ataxia right	1.0	0.0	1.0–1.0	1.0	0.0	1.0–1.0
15b	Limb ataxia left	0.66	0.31	0.04–1.0	−	−	−

Abbreviations: ASE, asymptotic standard error; LLE, left lower extremity; LOC, level of consciousness; LOC, loss of consciousness; LUE, left upper extremity; NOS-TBI, Neurological Outcome Scale for Traumatic Brain Injury; RLE, right lower extremity; RUE, right upper extremity.

## DISCUSSION

The NOS-TBI is the first clinical assessment tool for TBI patients that allows the systematic classification of neurological functions. 2 For the first time in the literature, we present the translated version of NOS-TBI in Brazilian Portuguese. Also, using standardized methodology in a representative sample of TBI patients, we show that the Brazilian Portuguese version of the NOS-TBI is a valid, sensitive to clinical change, and reproducible outcome measure for the assessment of TBI patients.

Here, interrater reliability was high and similar to those obtained in the original version, both for the global scale and subitems. 2 The internal consistency of NOS-TBI showed itself to be highly significant, expressed by an excellent value 5 and the Cronbach's α result found in this study was similar to that reported in the original NOS-TBI validation study, which was 0.94. It is important to note that the calculated “high value” α can be explained by the instrument's larger number of items. However, the analysis did not indicate redundancy between the items, and excluding an item did not represent a significant change in the α value.

The present study also confirmed the validity of concurrent criteria by finding statistically significant and strong correlations between our scale and the GCS and RTS. The two instruments used were different from those in the original scale. 3 However, this was justified since GCS is considered the gold standard for assessing the TBI population in Brazil and worldwide, and RTS is the score used in polytrauma patients. Additionally, the results corroborate the validation of the original instrument and suggest that NOS-TBI provides complementary and nonredundant information to the gold standard measurements.

As for convergent validity, the total score of the NOS-TBI was compared with the total score of FIM and GOS-E, resulting in a moderate correlation. Since NOS-TBI was designed to quantify specific TBI victims' deficits, 2 4 there are no other comparable instruments. Since convergent validity investigates whether there is a relationship between similar preexisting instruments, we would not expect convergent validity to present with the highest results. The correlation between NOS-TBI with FIM and GOS-E reviews how neurological function, evaluated by NOS-TBI, can affect aspects of an individual's global functional capacity. 16 Although the instruments did not provide identical evaluations, we suggest they all measured a similar neurological construction.

The validation of the NOS-TBI in Brazilian Portuguese has important implications for clinical practice in Brazil. A validated version of the scale can help standardize assessment, support prognostication, and guide rehabilitation strategies, ultimately contributing to improved patient care. Additionally, the availability of a validated NOS-TBI strengthens Brazil's participation in international research networks by enabling direct comparisons with global studies and facilitating inclusion in multinational collaborations. It may also support epidemiological research by helping to monitor TBI incidence and recovery trends nationwide. Overall, the cross-cultural validation of the NOS-TBI enhances Brazil's ability to contribute to high-quality multinational studies, advance TBI management, and inform global clinical guidelines. 17 18 19

This study has several limitations that should be considered when interpreting the results. The primary limitation is that it was conducted in a single center, which may limit the generalizability of the findings to other clinical or geographic contexts. The use of a convenience sample in the interrater reliability analysis may have introduced selection bias and affected the representativeness of the broader TBI patient population.

An additional limitation is that we did not evaluate the scale's applicability across different population subgroups. This is compounded by the fact that our sample—particularly at follow-up—consisted predominantly of individuals with mild TBI. Moreover, there was a gender imbalance in our sample, with men comprising 80.8%. However, the existing literature suggests that TBIs are more common in men due to higher exposure to risk factors such as traffic accidents, violence, and occupational hazards. 20 21

It is worth noting that we applied strict study methods following COSMIN guidelines, with prospective and systematically conducted data collection in a large cohort of individuals. This approach enabled us to accurately reflect the reality of the population treated at our institution. Future studies with a more diverse population can strengthen the validity and applicability of NOS-TBI in different levels of TBI severity, clinical and research settings.

The Brazilian Portuguese version of NOS-TBI adapted the scents from licorice and vanilla to coffee and banana to ensure conceptual equivalence and cultural relevance, thereby avoiding biases that could compromise the test's validity. 22 23 The cultural adaptation of translated scales in clinical research is crucial for several reasons. First, direct translation might not capture the intended meaning, leading to misinterpretation and inaccurate data. Second, to maintain validity and reliability, cross-cultural research must resonate with the cultural context of the target population. Lastly, participants are more likely to engage with a scale that reflects their cultural context. 24

Future studies should focus on integrating NOS-TBI into remote assessments using digital technologies to improve the evaluation of participants with TBI, similar to the recent use of the NIHSS for evaluating individuals after stroke. 25 26 With digital platforms, healthcare professionals will be able to apply the NOS-TBI remotely, conducting assessments efficiently even in geographically dispersed contexts. Further studies are needed, to evaluate the longitudinal validity, responsiveness to change, and utility of the NOS-TBI across diverse TBI populations in Brazil.

In conclusion, following the of cross-cultural adaptation and validation of this valuable instrument, the Brazilian Portuguese version of NOS-TBI can be considered a reliable and valid tool in the TBI setting. Furthermore, the structure of NOS-TBI makes it an appropriate convergent instrument, as it shows strong overlap with previously established tools. Overall, the NOS-TBI affirms itself as a reproducible instrument for assessing TBI in both the acute and chronic phases, in clinical practice and research.

Analysis of the psychometric properties of the Brazilian Portuguese NOS-TBI in this study revealed that the instrument is useful, valid, and reliable for assessing neurological function after a traumatic event, and more robust than commonly used scales, which often present several limitations. The development of relevant and reliable clinical scales in different languages, such as NOS-TBI, can facilitate multinational studies in the field.

**Figure 1:**
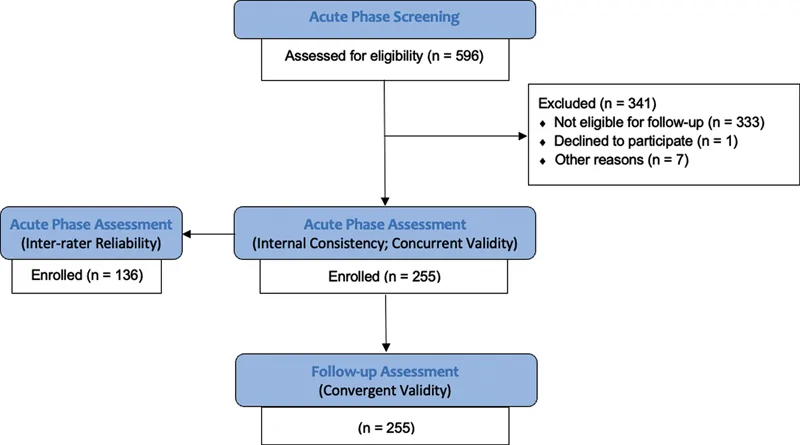
Note: The interrater reliability substudy, which is also depicted, consists of a convenience sample of participants enrolled in the acute phase. Flowchart of the recruitment process and inclusion/exclusion criteria. This diagram illustrates the systematic sampling used for the internal consistency and validity analyses.
